# Electrocardiographic (ECG) criteria for determining left ventricular mass in young healthy men; data from the *LARGE Heart *study

**DOI:** 10.1186/1532-429X-11-2

**Published:** 2009-01-16

**Authors:** Syed M Afzal Sohaib, John R Payne, Rajeev Shukla, Michael World, Dudley J Pennell, Hugh E Montgomery

**Affiliations:** 1 Centre for Cardiovascular Genetics, BHF Laboratories, Royal Free & University College Medical School, 5 University Street, London, UK; 2 Army Training Regiment Lichfield, Staffordshire, UK; 3 Royal Centre for Defence Medicine, Selly Oak Hospital, Birmingham, UK; 4 Cardiovascular Magnetic Resonance Unit, Royal Brompton Hospital, London, UK; 5 UCL Institute for Human Health and Performance, London, UK

## Abstract

**Background:**

Doubts remain over the use of the ECG in identifying those with increased left ventricular (LV) mass. This is especially so in young individuals, despite their high prevalence of ECG criteria for LV hypertrophy. We performed a study using cardiovascular magnetic resonance (CMR), which provides an *in vivo *non-invasive gold standard method of measuring LV mass, allowing accurate assessment of electrocardiography as a tool for defining LV hypertrophy in the young.

**Methods and results:**

Standard 12-lead ECGs were obtained from 101 Caucasian male army recruits aged (mean ± SEM) 19.7 ± 0.2 years. LV mass was measured using CMR. LV mass indexed to body surface area demonstrated no significant correlation with the Cornell Amplitude criteria or Cornell Product for LV hypertrophy. Moderate correlations were seen with the Sokolow-Lyon Amplitude (0.28) and Sokolow-Lyon Product (0.284). Defining LV hypertrophy as a body surface area indexed left ventricular mass of 93 g/m^2^, calculated sensitivities [and specificities] were as follows; 38.7% [74.3%] for the Sokolow-Lyon criteria, 43.4% [61.4%] for the Sokolow-Lyon Product, 19.4% [91.4%] for Cornell Amplitude, and 22.6% [85.7%] for Cornell Product. These values are substantially less than those reported for older age groups.

**Conclusion:**

ECG criteria for LV hypertrophy may have little value in determining LV mass or the presence of LV hypertrophy in young fit males.

## Background

Cardiovascular risk rises with increasing left ventricular (LV) mass [1], with dichotomously-defined LV hypertrophy a powerful and independent risk factor in a variety of disease states [2-4], and even amongst the healthy and normotensive [1,5]. Electrocardiography (ECG) has been used to help identify increased LV mass and thus cardiovascular risk[6,7]. However, doubts remain over the sensitivity and, increasingly, specificity of the ECG in identifying those with increased LV mass [6,8], especially in younger individuals [9-11]. Indeed, in 300 healthy post-pubertal boys (mean age 16 years) with a sedentary lifestyle, 23% fulfilled the Sokolow-Lyon Amplitude criteria for LV hypertrophy [10].

In an effort to resolve these issues, attempts have been made to define the sensitivity and specificity of ECG criteria in identifying LV hypertrophy, by comparison with echocardiographic estimates of LV mass [12-16]. However, few have attempted to address this issue in the young and fit [11,17]. Further, when applied to the individual, echocardiographic measures of LV mass are imprecise as they assume a uniform LV shape and apply a cubing mathematical formula to derive an estimation of LV mass. The advent of cardiovascular magnetic resonance (CMR) now offers a gold standard in the accurate quantification of LV mass [18], and studies confirm the weakness of echocardiography in this regard [19]. For this reason, CMR has been used to assess the validity of ECG-derived indices of LV mass; but to date, only in small cohorts of older adults [20,21].

Thus, we have used CMR to clarify whether the high prevalence of ECG-defined LV hypertrophy in the young truly represents the prevalence of LV hypertrophy. We have assessed four different criteria: the Sokolow-Lyon Amplitude [22], Cornell Amplitude[23], Sokolow-Lyon Product and Cornell Product [24] in diagnosing LV hypertrophy in a large sample of young male army recruits.

## Methods

This study complies with the Declaration of Helsinki and had appropriate ethics approval (Defence Medical Services Clinical Research Committee). Written informed consent was obtained from all participants.

### Study subjects

Subjects were enrolled in the LARGE Heart study, described in detail elsewhere [25]. In brief, consecutive healthy young Caucasian male army recruits were studied at entry to the Army Training Regiment, Lichfield, United Kingdom between July 2002 and April 2004. Any potential subjects with hypertension or regular medication use were excluded prior to the start of the study. Subject height and weight were recorded and body surface area (BSA) estimated using the Dubois formula: BSA = 0.20247 × height (m)^0.725 ^× weight (Kg)^0.425^. LV mass was quantified using CMR, and standard resting 12-lead electrocardiography was performed.

### CMR assessment of LV mass

Imaging was performed using a mobile 1.5 Tesla Siemens Sonata CMR scanner applying protocols previously described [26]. In brief, the LV short axis was identified by first piloting a vertical long axis (VLA) plane from the transaxial plane. The horizontal long axis (HLA) plane was imaged, and from this a stack of short axis (SA) images was obtained during breath-holding, covering the length of the LV. ECG-gated cine images were used in order to measure the LV mass at end-diastole. The temporal resolution was 21.6 ms. The in plane pixel size was 2.1 × 1.3 mm. All images were acquired using a steady state free precession (SSFP) sequence. Image analysis was performed by one investigator (JP) blind to other study data, using CMRtools (Cardiovascular Imaging Solutions, London, UK). The area of myocardium was calculated for each SA slice and, using Simpson's method, LV myocardial volume calculated. Trabeculae were included in the assessment of LV mass, but papillary muscles were excluded. This volume was then multiplied by myocardial tissue specific density (1.05 g/cm^3^). LV mass indexed to BSA was used in statistical analysis. All measurements of LV mass presented here were before a period of physical training and have been presented previously [25].

### Electrocardiography

Electrocardiographic assessment was performed immediately prior to scanning in a randomly-selected subgroup of individuals. A standard resting 12-lead recording was made during quiet respiration, with subjects in a supine position. The ECG was recorded at 25 mm/s and 0.1 mV/mm standardisation with a MAC 5000 resting ECG system (GE Medical Systems, UK). All ECG characteristics were determined from digital data using an appropriate software package (Cardiosoft, GE Medical Systems). From these measurements, one observer blinded to CMR data calculated 4 ECG criteria: Sokolow-Lyon Amplitude [22], Sokolow-Lyon Product [24], Cornell Amplitude [27] and Cornell Product [24]. LV hypertrophy was defined as a Sokolow-Lyon Amplitude of [SV_1 _+ RV_5 _or RV_6_] ≥ 35 mm; a Sokolow-Lyon Product of [(SV_1 _+ RV_5_) or (RV_6_) × QRS duration] ≥ 2940 mm·ms [20]; a Cornell Amplitude of [RaVL + SV_3_] ≥ 28 mm and a Cornell Product of [(RaVL + SV_3_) × QRS duration] ≥ 2440 mm·ms.

### Statistical analysis

Data were analysed using SPSS software (version 12.0.1). The relationship between LV mass indexed to BSA (indexed LV mass) and Sokolow-Lyon Amplitude, Sokolow-Lyon Product, Cornell Amplitude, and Cornell Product were assessed using Pearson's correlation coefficients. To assess the performance of different ECG criteria, LV hypertrophy was defined using three different criteria: The lowest threshold limit for 'acceptable' LV mass in this study was one defined using CMR in a study of 30 normal men, albeit of greater age (20–65 years) this being ≤ 83 g/m^2 ^[20,28]. However, 63% of our subjects had values of indexed LV mass greater than this. As a result, we studied two other thresholds; the first being 2 standard deviations (SD) above the mean for our group of subjects (108 g/m^2^). The other was based on a study that tried to define normal ranges according to age ranges. The lowest age range for males in that study was 20–29 and gave a cut off value of 93 g/m^2 ^2-SD above the mean [29]. Sensitivities and specificities for the existing cut off values for the Sokolow-Lyon Amplitude, Sokolow-Lyon Product, Cornell Amplitude, and Cornell Product were calculated for each of the three cut off values of LV hypertrophy. Performance of the different ECG criteria over the full range of amplitudes was assessed using receiver operator curve (ROC) characteristics. Curves were constructed using the three different cut off values for LV hypertrophy. Significance tests were performed to assess the difference between the area under the curve for the ROC curve and the area under the line of no discrimination (0.5). The mean indexed LV mass of the participants with and without ECG-defined LV hypertrophy were compared using an unpaired sample t-test.

## Results

One hundred and one subjects were enrolled, whose (mean ± SD) age was 19.7 ± 2.3 years, height 178.4 + 7.0 cm, weight 73.6 ± 9.8 kg, systolic blood pressure 122.1 ± 16.9 mm Hg, diastolic blood pressure 68.4 ± 10.5 mm Hg, LV mass 168.0 ± 24.9 g and LV mass indexed to BSA 87.7 ± 10.3 g/m^2^. Prevalence of ECG-defined LV hypertrophy was 29.7%, 41.6%, 11.9% and 16.8% for Sokolow-Lyon Amplitude, Sokolow-Lyon Product, Cornell Amplitude and Cornell Product criteria respectively.

We assessed the correlation of Sokolow-Lyon Amplitude, Sokolow-Lyon Product, Cornell Amplitude and Cornell Product with LV mass index (Table 1). A significant association was only demonstrable with the Sokolow-Lyon Amplitude and Product (R = 0.280 and 0.284 respectively).

**Table 1:** Correlation with LV mass indexed to BSA

**Criteria**	**Pearson Correlation Coefficient**	**P-value**
Sokolow-Lyon Amplitude (mm)	0.280	0.005
Sokolow-Lyon Product (mm·ms)	0.284	0.004
Cornell Amplitude (mm)	0.065	0.516
Cornell Product (mm·ms)	0.097	0.336

**Figure 1:**
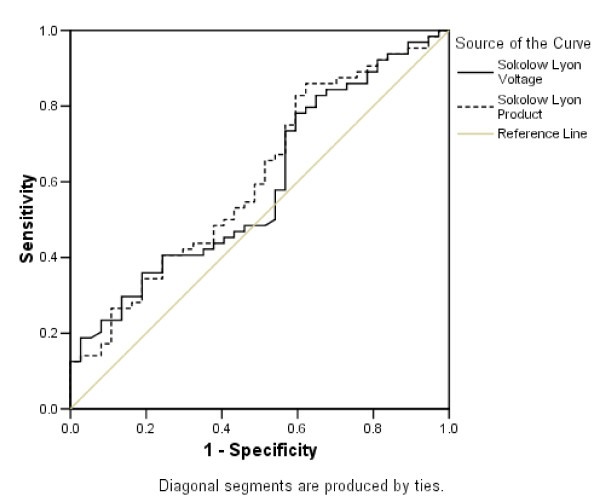
**ROC curves with a cut off value of 83 g/m^2 ^for LV hypertrophy**.

**Figure 2:**
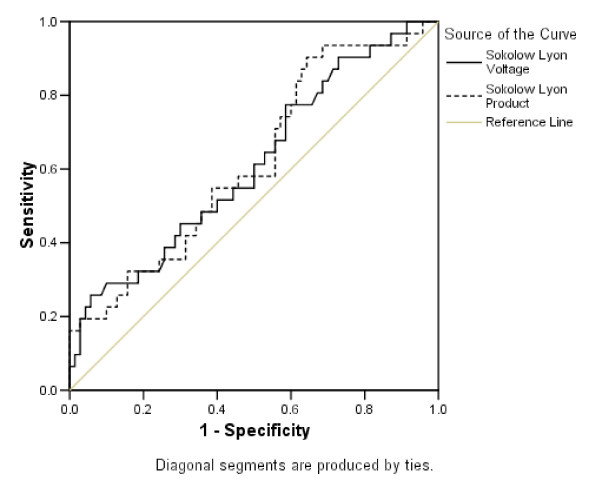
**ROC curves with a cut off value of 93 g/m^2 ^for LV hypertrophy**.

**Figure 3:**
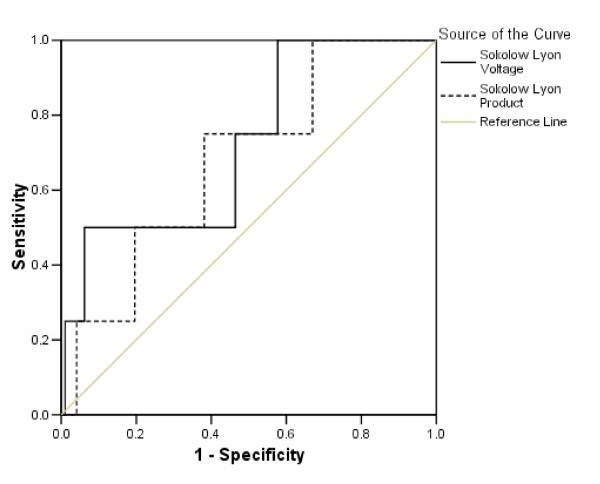
**ROC curves with a cut off value of 108 g/m^2 ^for LV hypertrophy**.

Sensitivity and specificity of the four ECG criteria in identifying CMR-determined LV hypertrophy was then addressed using 83 g/m^2 ^as a threshold for LV hypertrophy (Table 2). All four ECG criteria demonstrate low sensitivity (14.1% to 43.8%) with variable specificities (62.2% to 91.9%). The sensitivity of these criteria is not impressive at the threshold of 93 g/m^2 ^(sensitivity from 19.4% to 43.4% and specificity from 61.4% to 91.4%). On moving the threshold up to 108 g/m^2^, sensitivity improves (up to 75% for the Sokolow-Lyon criteria) with a further fall in specificity (59.8% to 88.7%). The prevalence of LV hypertrophy for the cut off values was 63% at 83 g/m2, 31% at 93 g/m2, and 4% at 108 g/m2. ROC curves for the Sokolow-Lyon criteria were constructed using the three different cut off values for LV hypertrophy (Figures 1, 2 and 3). There was no statistically significant difference between the area under the curve for any of the amplitude criteria and the line of no discrimination (Table 3).

**Table 2:** Sensitivities and specificities according to three partitions values of LV mass indexed to BSA (sens = sensitivity, spec = specificity).

	**LV mass indexed to BSA partition values**					
	**83 g/m**^**2**^		**93 g/m**^**2**^		**108 g/m**^**2**^	

*ECG Criteria*	*Sens* *(%)*	*Spec* *(%)*	*Sens* *(%)*	*Spec* *(%)*	*Sens* *(%)*	*Spec* *(%)*

Sokolow-Lyon Amplitude >35 mm	35.9	*81.1*	38.7	*74.3*	50.0	*71.1*
Sokolow-Lyon Product >2940 mm·ms	43.8	*62.2*	43.4	*61.4*	75.0	*59.8*
Cornell Amplitude >28 mm	14.1	*91.9*	19.4	*91.4*	25.0	*88.7*
Cornell Product >2440 mm·ms	18.8	*86.5*	22.6	*85.7*	25.0	*83.5*

**Table 3:** Area under curve values from ROC curve analysis.

	**Area Under Curve (significance)**		
**LVH Cut off Value:**	**83 g/m**^**2**^	**93 g/m**^**2**^	**108 g/m**^**2**^

Sokolow-Lyon Amplitude	0.592 (0.13)	0.613 (0.07)	0.722 (0.13)

Sokolow-Lyon Product	0.610 (0.07)	0.618 (0.06)	0.678 (0.23)

Cornell Amplitude	0.494 (0.92)	0.524 (0.71)	0.469 (0.83)

Cornell Product	0.514 (0.81)	0.533 (0.60)	0.454 (0.75)

Finally, we calculated the mean LV mass index for those subjects categorised as having or lacking LV hypertrophy as defined by each of the four ECG criteria (Table 4). Only using the Sokolow-Lyon Amplitude was there a significant difference in LV mass index between ECG positive and ECG negative.

**Table 4:** Mean LV mass index for subjects categorized as having or lacking LV hypertrophy (LVH).

	**LV mass indexed to BSA (g/m^**2**^)** **Mean ± SD (n)**		
**Criteria**	**ECG: No LVH**	**ECG: LVH**	**P-value**

Sokolow-Lyon Amplitude >35 mm	86.2 ± 9.9 (71)	91.3 ± 10.4 (30)	0.023

Sokolow-Lyon Product >2940 mm·ms	86.4 ± 9.5 (59)	89.6 ± 11.1 (42)	0.126

Cornell Amplitude >28 mm	87.1 ± 10.1 (89)	92.4 ± 10.5 (12)	0.092

Cornell Product >2440 mm·ms	87.2 ± 10.2 (84)	90.3 ± 10.3 (17)	0.250

## Discussion

This is the first study to examine the validity of electrocardiography in the determination of LV hypertrophy in young healthy males, using CMR-measured (as opposed to echocardiographically-imputed) LV mass as the gold-standard. It demonstrates the poor sensitivity of established ECG criteria in identifying LV hypertrophy in such individuals.

Our data confirm the high incidence of electrocardiographically-defined LV hypertrophy amongst the young and healthy. Twenty-nine percent had LV hypertrophy as defined by Sokolow-Lyon ECG Amplitude criteria, a figure similar to the 23% incidence previously observed in healthy teenage non-athletes [10]. One would expect the incidence of physiological left ventricular hypertrophy to be higher in this cohort as army recruits will be more athletic than the general population. Other studies have showed a higher prevalence of ECG defined left ventricular hypertrophy in the athletic rising to 50% amongst 172 teenage soccer players [11], and 45% amongst 1000 teenage elite athletes [10].

The amplitude criteria assessed here were all derived using various markers to indicate LV hypertrophy, but all in older age groups, and before the advent of CMR. Echocardiography or autopsy were used to define or validate the criteria tested here, and in the case of the Sokolow Lyon, the criteria were based on clinical features predominantly [30]. Most of the subjects in these validation studies would have had pathological LV hypertrophy, unlike in this study.

The CMR data presented here shows some striking differences to the validation studies, and other studies assessing ECG criteria for LV hypertrophy. Across a range of past studies and methods, we see generally poor sensitivities, but relatively good specificities, with correlation coefficients clustering between 0.5 and 0.6, but these have all been done in older subjects, many of whom have established cardiac pathology [23,27,24,11,16]. The correlation coefficients are much weaker in this cohort despite similar sample sizes to older studies. Only the Sokolow-Lyon Amplitude and Product show a significant correlation with indexed LV mass, and even this is weak. No relationship is seen with the Cornell Amplitude or Cornell Product. This implies that in a population of young fit males, existing ECG amplitude criteria do little to indicate LV mass. Of the existing amplitude criteria, the Sokolow-Lyon Amplitude and Product may give some indication of LV mass, but much less so than in a group of older adults with other evidence of underlying cardiac disease. It is unclear why the Sokolow Lyon criteria should show a stronger relationship in the young to the Cornell criteria. There is likely to be a feature in this younger cohort which diminish the use of the Cornell criteria, such as body habitus. The BSA measurements confirm that the individuals in this study are very thin chested which would impact on the specificity of some of the ECG criteria. One could speculate that the Cornell criteria are better at detecting pathological left ventricular hypertrophy rather than physiological hypertrophy which is likely to be the predominant form in this group and hence these results. According to these results, while the Sokolow Lyon criteria may give some indication of LV mass, they are not likely to demonstrate whether this is pathological, and is unlikely to be of any clinical use in this age group.

Some recent studies have tried to establish normal ranges for LV mass stratified by age group and sex. The cut off used in this study used an indexed LV mass cut off of 83 g/m^2^, based on a study of 60 men with a mean age of 43 years[28]. A more recent study has tried to define indexed LV mass values using SSFP CMR stratified by age and sex [29]. In this study an upper limit of 93 g/m^2 ^is inferred for men aged 20–29. This was based on a value 2 standard deviations (SD) above the mean from a sample of 10 men aged 20–29 years. That study did not include those aged under 20 years, the age of many of the participants in our report but for the purposes of this study provided a reasonable approximation. Unfortunately most of these estimates are based on small cohorts. In this cohort, the value 2-SD above the upper limit of normal was 108 g/m^2^, which is much higher than either of the quoted figures. Participants in this study are army recruits and may be more active than their peers, so this higher cut off may represent an element of physiological LV hypertrophy seen in athletes [11,17]. In fact in the analysis, there was little evidence of pathological forms of LV hypertrophy such as hypertrophic cardiomyopathy, so most of the increased LV mass is likely to be physiological in this cohort.

Despite the absence of an established mass cut off in this age group, across the three mass cut-offs for LV hypertrophy, the ROC curves demonstrate that all the ECG criteria are poor at delineating LV hypertrophy. One pays the price either with sensitivity or specificity over a range of amplitudes. With none of the cut off values for indexed LV mass was there a statistically significant difference between the line of no discrimination.

We did show that participants who were Sokolow-Lyon Amplitude positive for LV hypertrophy did have a higher LV mass, but this is not surprising considering the slight positive correlation between indexed LV mass and Sokolow-Lyon Amplitude. This must, however, be interpreted in the context of the ROC curves for these criteria, which imply there is little practical use for the Sokolow-Lyon Amplitude in the diagnosis of LV hypertrophy in the young.

There are a number of strengths to this study. It has used CMR rather than echocardiography to determine LV mass. It has used this gold standard to validate and assess these commonly used ECG criteria in population of fit young males unlike the majority of previous work, which has been in an older population, who often have disease. There are, however, some limitations. The sample is relatively small, and the subjects are all male. It is difficult to perform such a study when there is no well-established cut off for normal LV mass in young males. This became particularly apparent when analysing the participants who had an LV mass 2 SD above the mean for this group where only four subjects were present. In such a population increased LV mass in itself may be marker of health rather than disease. The population is white and does not account for racial differences [31].

To conclude, commonly used amplitude criteria may have little practical use in detecting LV hypertrophy in the young. In particular, Cornell Amplitude and Cornell Product have little relationship with LV mass in a group of young males. Whilst the Sokolow-Lyon Amplitude has a (weak) correlation with LV mass, the price is paid in terms of specificity and sensitivity when more stringent criteria defining LVH are applied.

## Competing interests

The authors declare that they have no competing interests.

## Authors' contributions

HEM and JRP conceived the study. JRP collected electrocardiographic data with support from MW. Numerical data were extracted and analysed by SMAS and RS. DP oversaw MRI acquisition and analysis. All authors contributed to the preparation of the manuscript, and read and approved the final manuscript.

## References

[B1] Levy D, Garrison RJ, Savage DD, Kannel WB, Castelli WP (1990). Prognostic implications of echocardiographically determined left ventricular mass in the Framingham Heart Study. N Engl J Med.

[B2] Koren MJ, Devereux RB, Casale PN, Savage DD, Laragh JH (1991). Relation of left ventricular mass and geometry to morbidity and mortality in uncomplicated essential hypertension. Ann Intern Med.

[B3] Ghali JK, Liao Y, Simmons B, Castaner A, Cao G, Cooper RS (1992). The prognostic role of left ventricular hypertrophy in patients with or without coronary artery disease. Ann Intern Med.

[B4] Bolognese L, Dellavesa P, Rossi L, Sarasso G, Bongo AS, Scianaro MC (1994). Prognostic value of left ventricular mass in uncomplicated acute myocardial infarction and one-vessel coronary artery disease. Am J Cardiol.

[B5] Post WS, Levy D (1994). New developments in the epidemiology of left ventricular hypertrophy. Curr Opin Cardiol.

[B6] Levy D, Labib SB, Anderson KM, Christiansen JC, Kannel WB, Castelli WP (1990). Determinants of sensitivity and specificity of electrocardiographic criteria for left ventricular hypertrophy. Circulation.

[B7] Messerli FH, Aepfelbacher FC (1995). Hypertension and left-ventricular hypertrophy. Cardiol Clin.

[B8] Devereux RB, Koren MJ, de SG, Okin PM, Kligfield P (1993). Methods for detection of left ventricular hypertrophy: application to hypertensive heart disease. Eur Heart J.

[B9] Edhouse J, Thakur RK, Khalil JM (2002). ABC of clinical electrocardiography. Conditions affecting the left side of the heart. BMJ.

[B10] Sharma S, Whyte G, Elliott P, Padula M, Kaushal R, Mahon N, McKenna WJ (1999). Electrocardiographic changes in 1000 highly trained junior elite athletes. Br J Sports Med.

[B11] Somauroo JD, Pyatt JR, Jackson M, Perry RA, Ramsdale DR (2001). An echocardiographic assessment of cardiac morphology and common ECG findings in teenage professional soccer players: reference ranges for use in screening. Heart.

[B12] Gasperin CA, Germiniani H, Facin CR, Souza AM, Cunha CL (2002). An analysis of electrocardiographic criteria for determining left ventricular hypertrophy. Arq Bras Cardiol.

[B13] Schillaci G, Verdecchia P, Borgioni C, Ciucci A, Guerrieri M, Zampi I, Battistelli M, Bartoccini C, Porcellati C (1994). Improved electrocardiographic diagnosis of left ventricular hypertrophy. Am J Cardiol.

[B14] Norman JE, Levy D (1995). Improved electrocardiographic detection of echocardiographic left ventricular hypertrophy: results of a correlated data base approach. J Am Coll Cardiol.

[B15] Norman JE, Levy D, Campbell G, Bailey JJ (1993). Improved detection of echocardiographic left ventricular hypertrophy using a new electrocardiographic algorithm. J Am Coll Cardiol.

[B16] Okin PM, Roman MJ, Devereux RB, Kligfield P (1995). Electrocardiographic identification of increased left ventricular mass by simple voltage-duration products. J Am Coll Cardiol.

[B17] Raskoff WJ, Goldman S, Cohn K (1976). The "athletic heart". Prevalence and physiological significance of left ventricular enlargement in distance runners. JAMA.

[B18] Myerson SG, Bellenger NG, Pennell DJ (2002). Assessment of left ventricular mass by cardiovascular magnetic resonance. Hypertension.

[B19] ellenger NG, Burgess MI, Ray SG, Lahiri A, Coats AJ, Cleland JG, Pennell DJ (2000). Comparison of left ventricular ejection fraction and volumes in heart failure by echocardiography, radionuclide ventriculography and cardiovascular magnetic resonance; are they interchangeable?. Eur Heart J.

[B20] Alfakih K, Walters K, Jones T, Ridgway J, Hall AS, Sivananthan M (2004). New gender-specific partition values for ECG criteria of left ventricular hypertrophy: recalibration against cardiac MRI. Hypertension.

[B21] Carlsson MB, Trägårdh E, Engblom H, Hedström E, Wagner G, Pahlm O, Arheden H (2006). Left ventricular mass by 12-lead electrocardiogram in healthy subjects: comparison to cardiac magnetic resonance imaging. J Electrocardiol.

[B22] Sokolow M, Lyon TP (2001). The ventricular complex in left ventricular hypertrophy as obtained by unipolar precordial and limb leads. 1949. Ann Noninvasive Electrocardiol.

[B23] Casale PN, Devereux RB, Kligfield P, Eisenberg RR, Miller DH, Chaudhary BS, Phillips MC (1985). Electrocardiographic detection of left ventricular hypertrophy: development and prospective validation of improved criteria. J Am Coll Cardiol.

[B24] Molloy TJ, Okin PM, Devereux RB, Kligfield P (1992). Electrocardiographic detection of left ventricular hypertrophy by the simple QRS voltage-duration product. J Am Coll Cardiol.

[B25] Payne JR, Eleftheriou KI, James LE, Hawe E, Mann J, Stronge A, Kotwinski P, World M, Humphries SE, Pennell DJ, Montgomery HE (2006). Left ventricular growth response to exercise and cigarette smoking: data from LARGE Heart. Heart.

[B26] Bellenger NG, Davies LC, Francis JM, Coats AJ, Pennell DJ (2000). Reduction in sample size for studies of remodeling in heart failure by the use of cardiovascular magnetic resonance. J Cardiovasc Magn Reson.

[B27] Casale PN, Devereux RB, Alonso DR, Campo E, Kligfield P (1987). Improved sex-specific criteria of left ventricular hypertrophy for clinical and computer interpretation of electrocardiograms: validation with autopsy findings. Circulation.

[B28] Alfakih K, Plein S, Thiele H, Jones T, Ridgway JP, Sivananthan MU (2003). Normal human left and right ventricular dimensions for MRI as assessed by turbo gradient echo and steady-state free precession imaging sequences. J Magn Reson Imaging.

[B29] Maceira AM, Prasad SK, Khan M, Pennell DJ (2006). Normalized left ventricular systolic and diastolic function by steady state free precession cardiovascular magnetic resonance. J Cardiovasc Magn Reson.

[B30] Sokolow M, Freidlander RD (1949). The normal unipolar precordial and limb lead electrocardiogram. Am Heart J.

[B31] Rautaharju PM, Park LP, Gottdiener JS, Siscovick D, Boineau R, Smith V, Powe NR (2000). Race- and sex-specific ECG models for left ventricular mass in older populations. Factors influencing overestimation of left ventricular hypertrophy prevalence by ECG criteria in African-Americans. J Electrocardiol.

